# Supplementary material for: Hidden in plain sight—highly abundant and diverse planktonic freshwater Chloroflexi
Source: Microbiome. 2018 Oct 2;6:176. doi: 10.1186/s40168-018-0563-8 (PMC6169038; doi:10.1186/s40168-018-0563-8)
Supplement: Supplementary file 1 — Figure S1. Maximum likelihood 16S rRNA tree reconstructed by adding the 16S rRNA sequences assembled from freshwater metagenomes to existing sequences of the SSURef_NR99_128 database in the phylum Chloroflexi. Figure S2. Percentage and distribution of Chloroflexi-related 16S rRNA reads (as % of total prokaryotic community) based on unassembled metagenomic datasets in brackish and marine datasets. Figure S3. Vertical profiles of water temperature, oxygen, phytoplankton and absolute CARD-FISH abundances of three lineages of Chloroflexi in Lake Zurich at five different sampling point in 2015. Figure S4. Vertical profiles of water temperature, oxygen, phytoplankton and absolute CARD-FISH abundances of three lineages of Chloroflexi in Rimov Reservoir at four different sampling points in 2015. Figure S5. Recruitment plot for ZSMay80m-G1 as a representative of the Chloroflexi CL500-11 cluster against different freshwater environments and the depth profile of brackish Caspian Sea. Figure S6. Recruitment plot for ZSMar2m-G89 as a representative of the Chloroflexi SL56 cluster against different freshwater environments. Figure S7. Recruitment plot for ZSMay80m-G79 as a representative of the Chloroflexi TK10 cluster against deep Caspian Sea dataset and different freshwater environments. Figure S8. Maximum likelihood tree of rhodopsin protein sequences from different bacterial and archaeal groups. Figure S9. Maximum likelihood tree of the pufM protein sequences from different bacterial groups. (PDF 3016 kb) [file 40168_2018_563_MOESM1_ESM.pdf]

Hidden in plain sight - highly abundant and diverse planktonic freshwater *Chloroflexi*

Maliheh Mehrshad[1*], Michaela M. Salcher[2], Yusuke Okazaki[3], Shin-ichi Nakano[3], Karel Šimek[1], Adrian-Stefan Andrei[1], Rohit Ghai[1*]

[1] Biology Centre of the Czech Academy of Sciences, Institute of Hydrobiology, Department of Aquatic Microbial Ecology, České Budějovice, Czech Republic

[2] Department of Limnology, Institute of Plant Biology, University of Zurich, Seestrasse 187, CH-8802 Kilchberg, Switzerland

[3] Center for Ecological Research, Kyoto University, 2-509-3 Hirano, Otsu, Shiga, 520-2113, Japan

*Corresponding authors:

Maliheh Mehrshad

Rohit Ghai

Institute of Hydrobiology, Department of Aquatic Microbial Ecology, Biology Centre ASCR

Na Sádkách 7, 370 05, České Budějovice, Czech Republic

Tel: 00420 38777 5819

Email: chaji.ml@gmail.com,

ghai.rohit@gmail.com

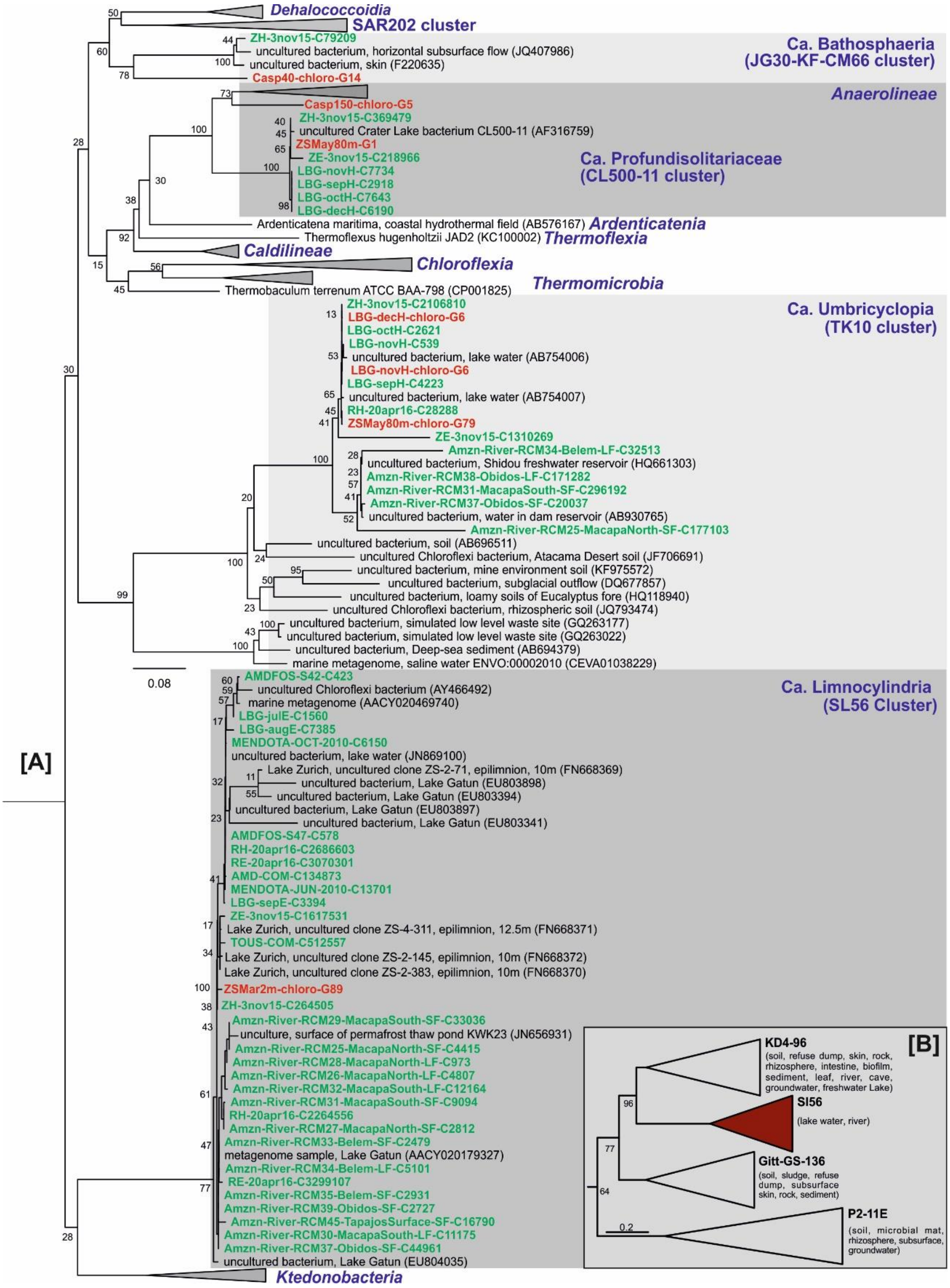

Supplementary Figure S1- Maximum likelihood 16S rRNA tree reconstructed by adding the 16S rRNA sequences assembled from freshwater metagenomes to existing sequences of the SSURef_NR99_128 database in the phylum *Chloroflexi*. Bootstrap values (%) are indicated at the base of each node. 16S rRNA sequences present in a MAG are highlighted in red and the other metagenomic assembled 16S rRNA sequences are highlighted in green [A]. Maximum likelihood 16S rRNA tree of the SL56 cluster together with its closely related clusters. The origin of the 16S rRNA sequences present in SILVA for each cluster are summarized in parenthesis [B].

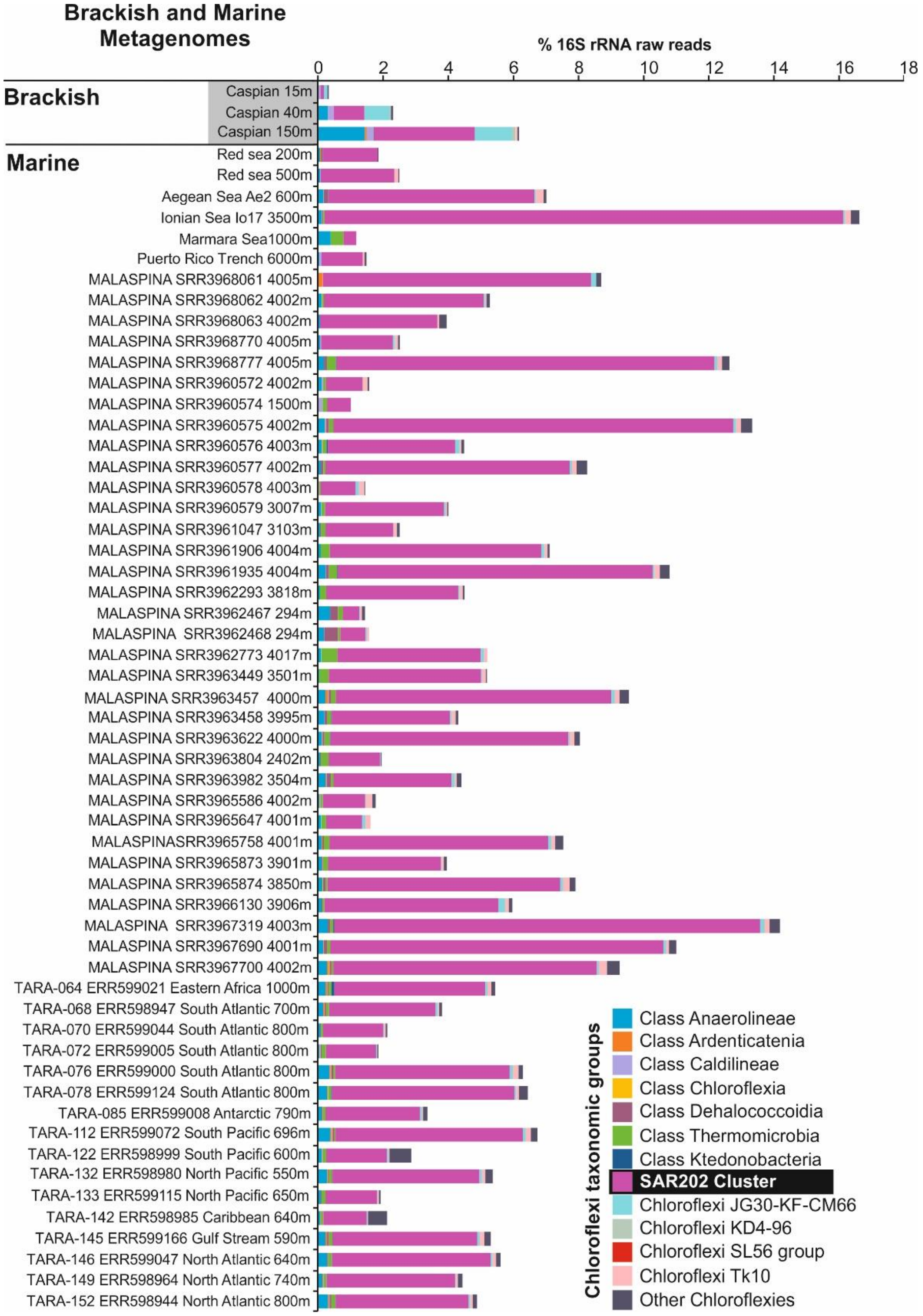

Supplementary Figure S2- Percentage and distribution of *Chloroflexi* related 16S rRNA reads (as % of total prokaryotic community) based on unassembled metagenomic datasets in brackish and marine datasets. Brackish datasets include three different depths of the Caspian Sea. Marine datasets include Aegean Sea (one DCM and one deep dataset), Ionian Sea (one DCM and one deep dataset), Atlantic BATS, Pacific HOTS and Red Sea depth profile datasets together with selected deep datasets from MALASPINA and TARA expeditions and the Puerto Rico deep trench dataset. Chloroflexi related reads were further assigned to lower taxonomic levels of the phylum Chloroflexi based on the best BLAST hit to class-level taxa. The complete list of datasets used is available in (Mehrshad *et al.*, 2017). Datasets highlighted in gray were used for the assembly.

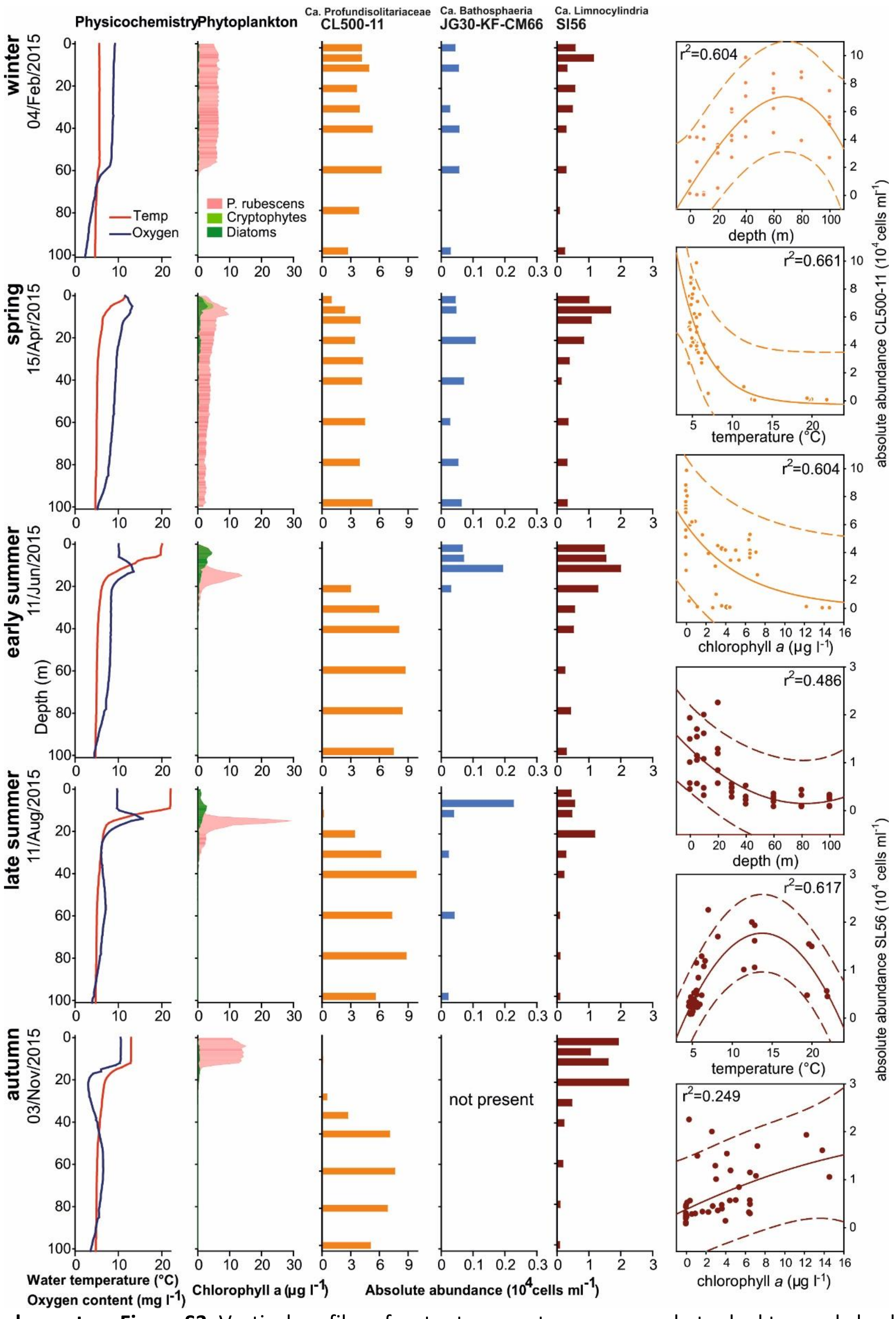

Supplementary Figure S3: Vertical profiles of water temperature, oxygen,phytoplankton and absolute CARD-FISH abundances of three lineages of Chloroflexi in Lake Zurich at five different sampling point in 2015. Relationships of absolute abundances of the CL500-11 and SL56 groups to depth, temperature and chlorophyll *a* are shown at the right. Correlation coefficients (r^2) are indicated within the plots.

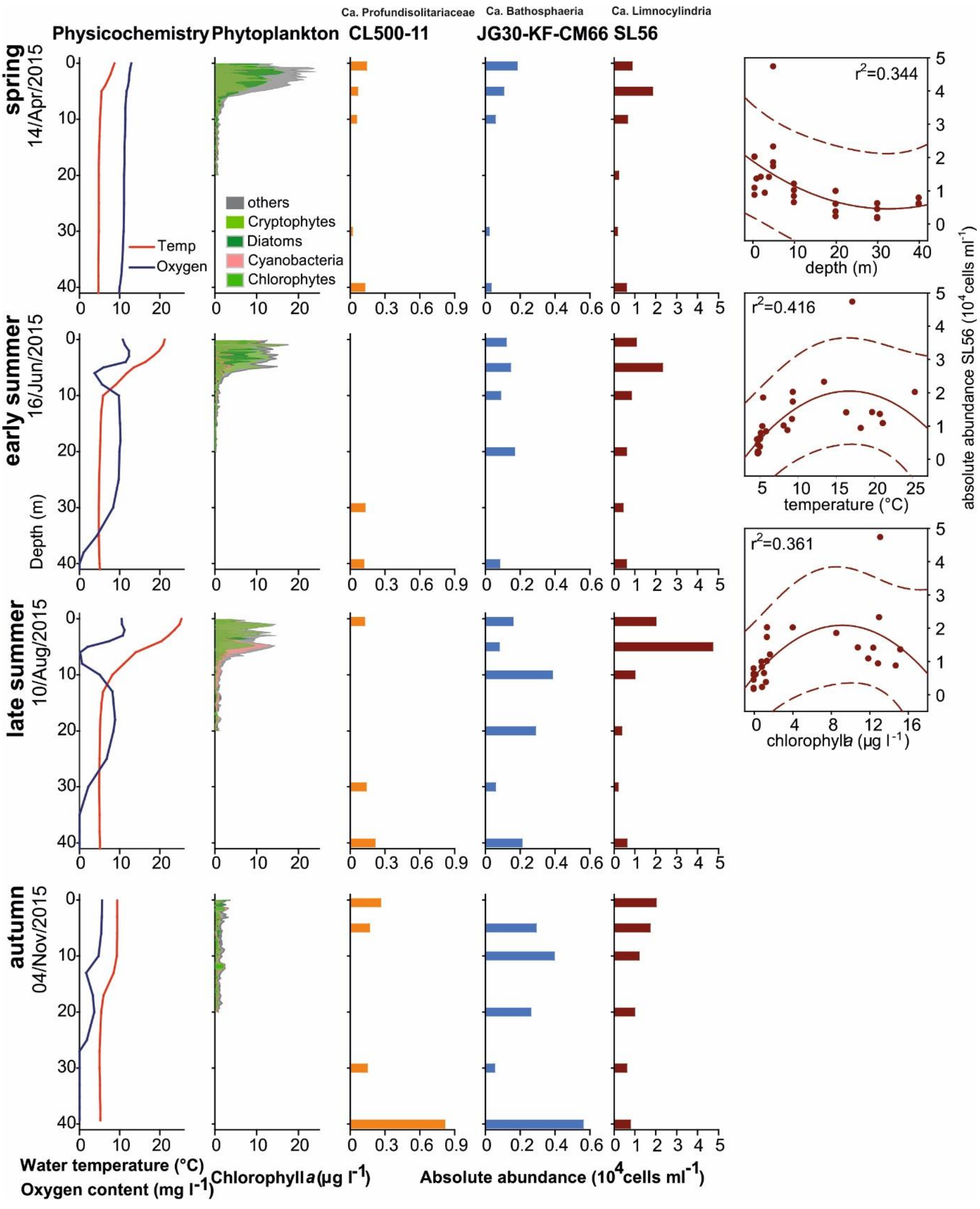

Supplementary Figure S4: Vertical profiles of water temperature, oxygen, phytoplankton and absolute CARD-FISH abundances of three lineages of Chloroflexi in Rimov Reservoir at four different sampling points in 2015. Relationships of absolute abundance of the SL56 group to depth, temperature and chlorophyll *a* are shown at the right. Correlation coefficients (r^2) are indicated within the plots.

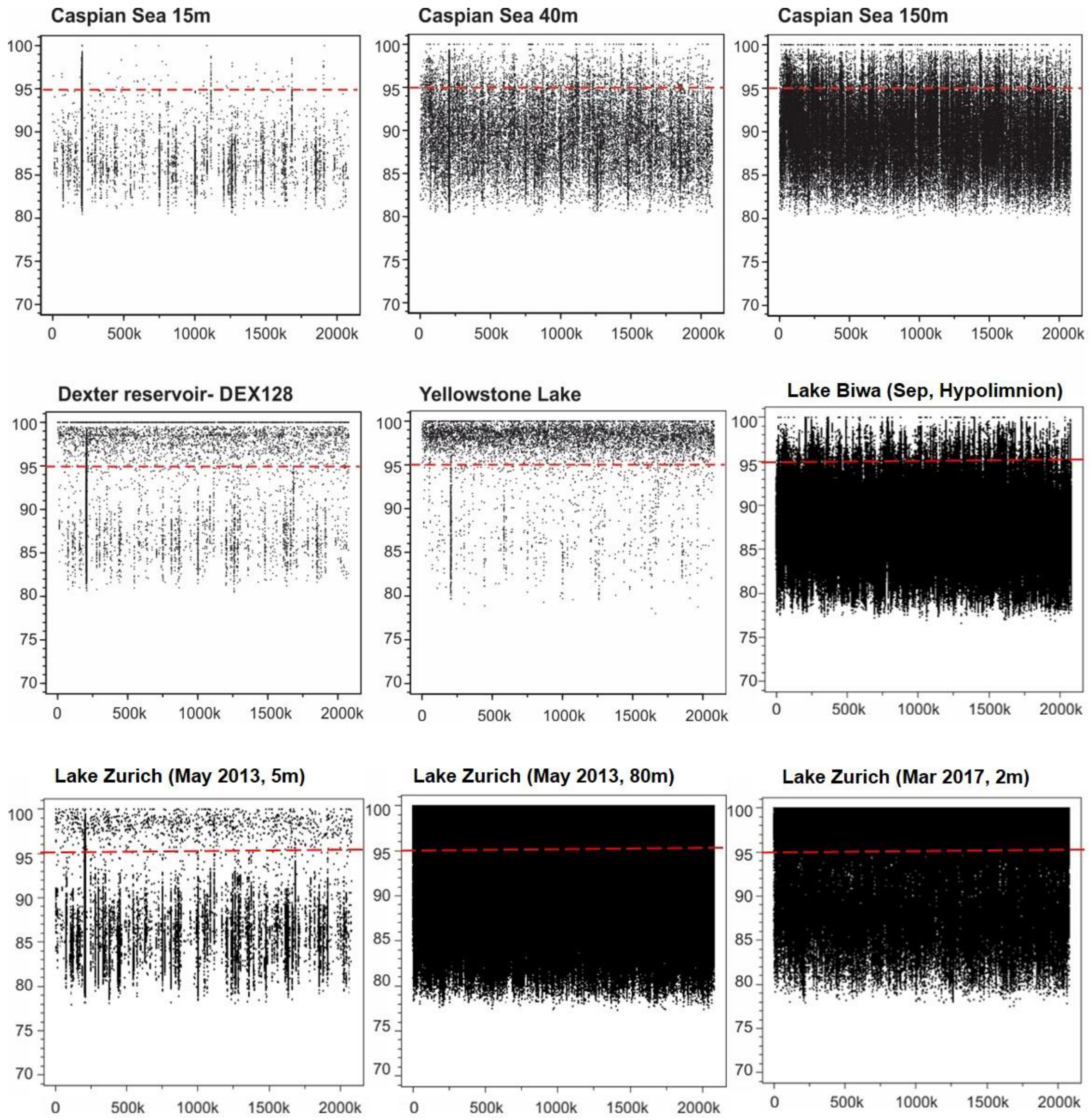

Supplementary Figure S5: Recruitment plot for ZSMay80m-G1 as a representative of the *Chloroflexi* CL500-11 cluster against different freshwater environments and the depth profile of brackish Caspian Sea. The ZSMay80m-G1 is the only bin that contains a 16S rRNA sequence and shows completeness of 75%. In each panel the Y axis represents the identity percentage and X axis represents the genome length. The red dashed line shows the threshold for presence of same species (95% identity).

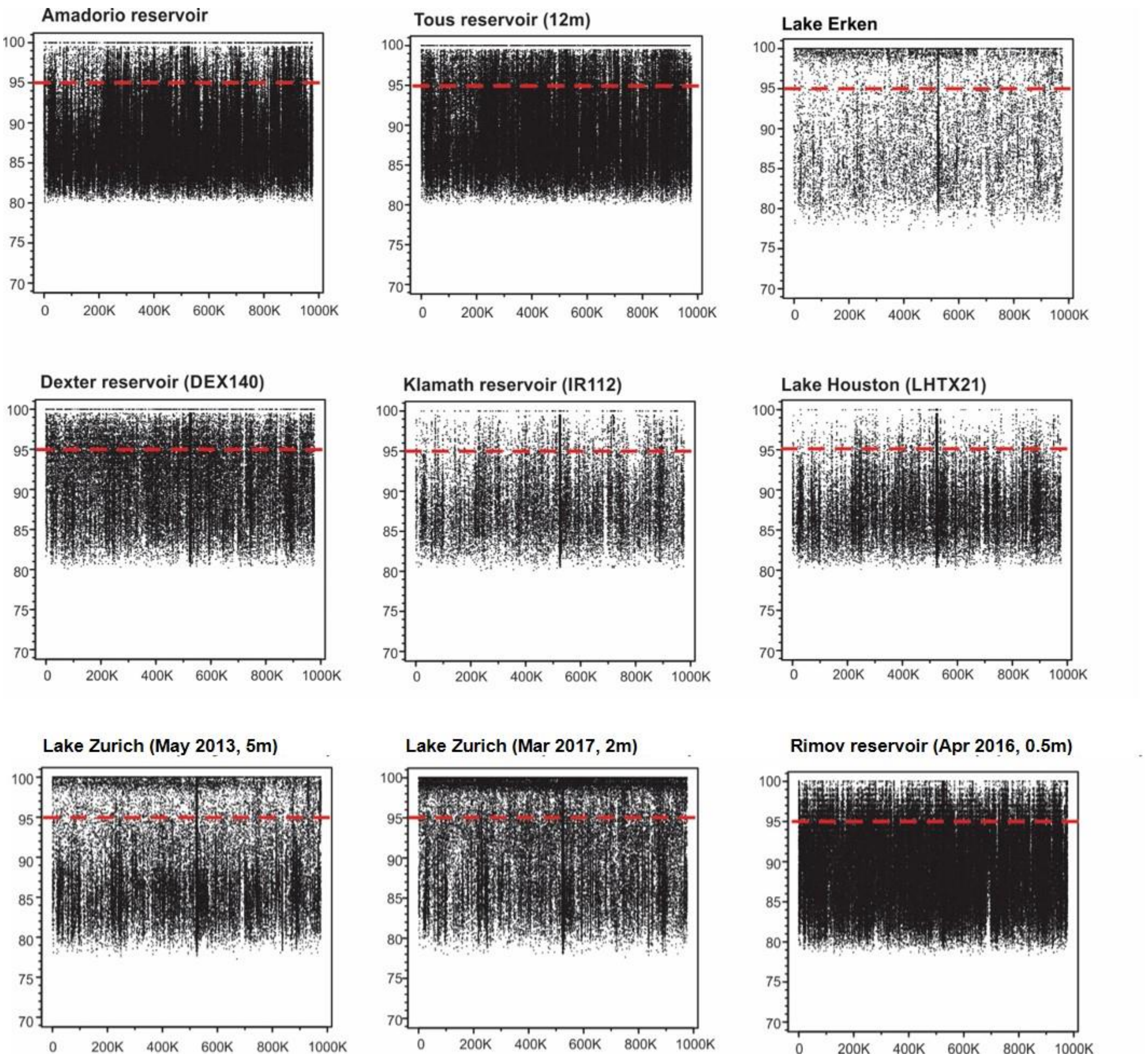

Supplementary Figure S6: Recruitment plot for ZSMar2m-G89 as a representative of the *Chloroflexi* SL56 cluster against different freshwater environments. The ZSMar2m-G89 is the only bin that contains a 16S rRNA sequence and shows completeness of 68%. In each panel the Y axis represents the identity percentage and X axis represents the genome length. The red dashed line shows the threshold for presence of same species (95% identity).

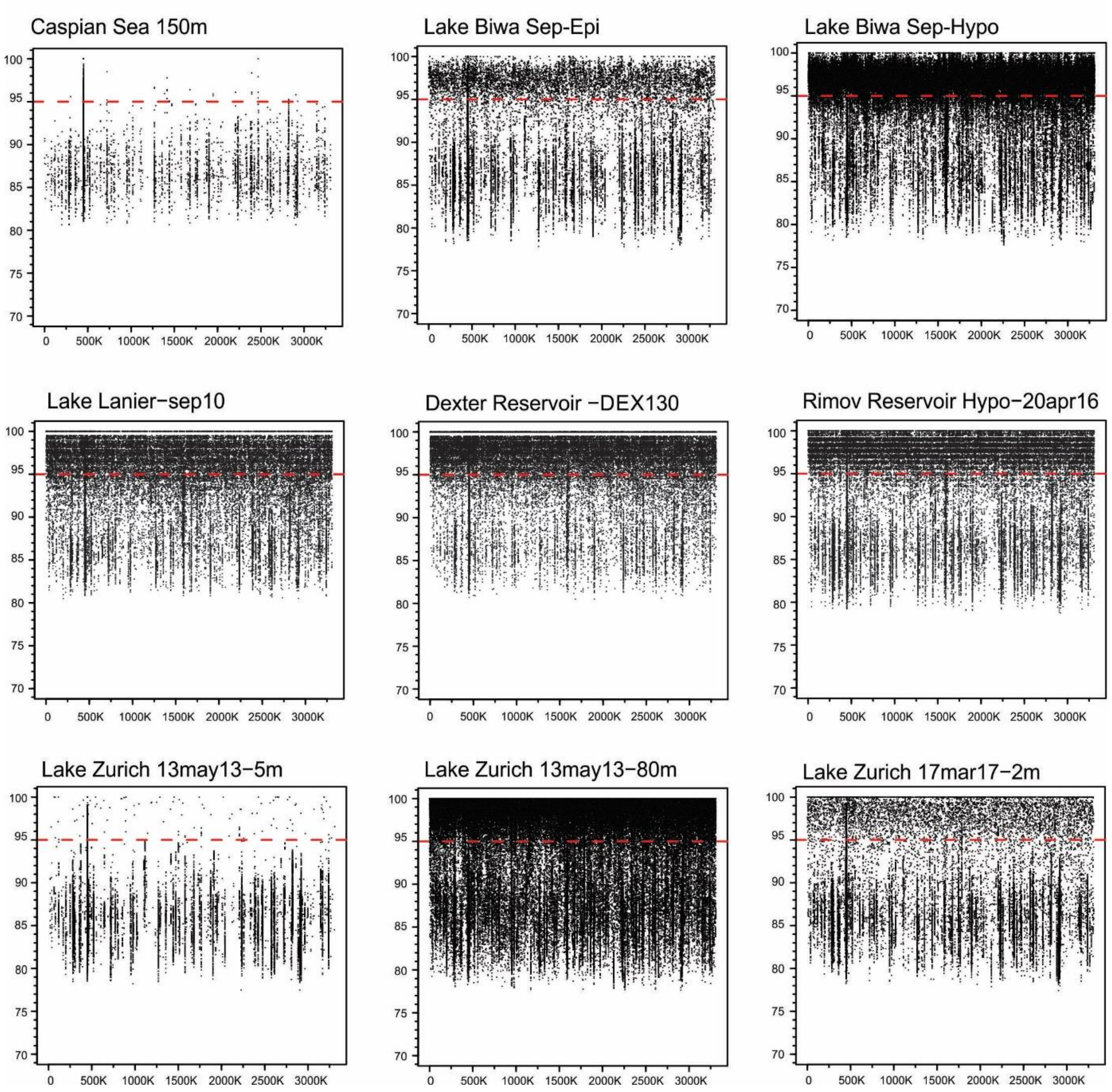

Supplementary Figure S7: Recruitment plot for ZSMay80m-G79 as a representative of the Chloroflexi TK10 cluster against deep Caspian Sea dataset and different freshwater environments. The ZSMay80m-G79 is the most complete genome in the TK10 cluster (85%) and also contains a 16S rRNA sequence. In each panel the Y axis represents the identity percentage and X axis represents the genome length. The red dashed line shows the threshold for presence of same species (95% identity).

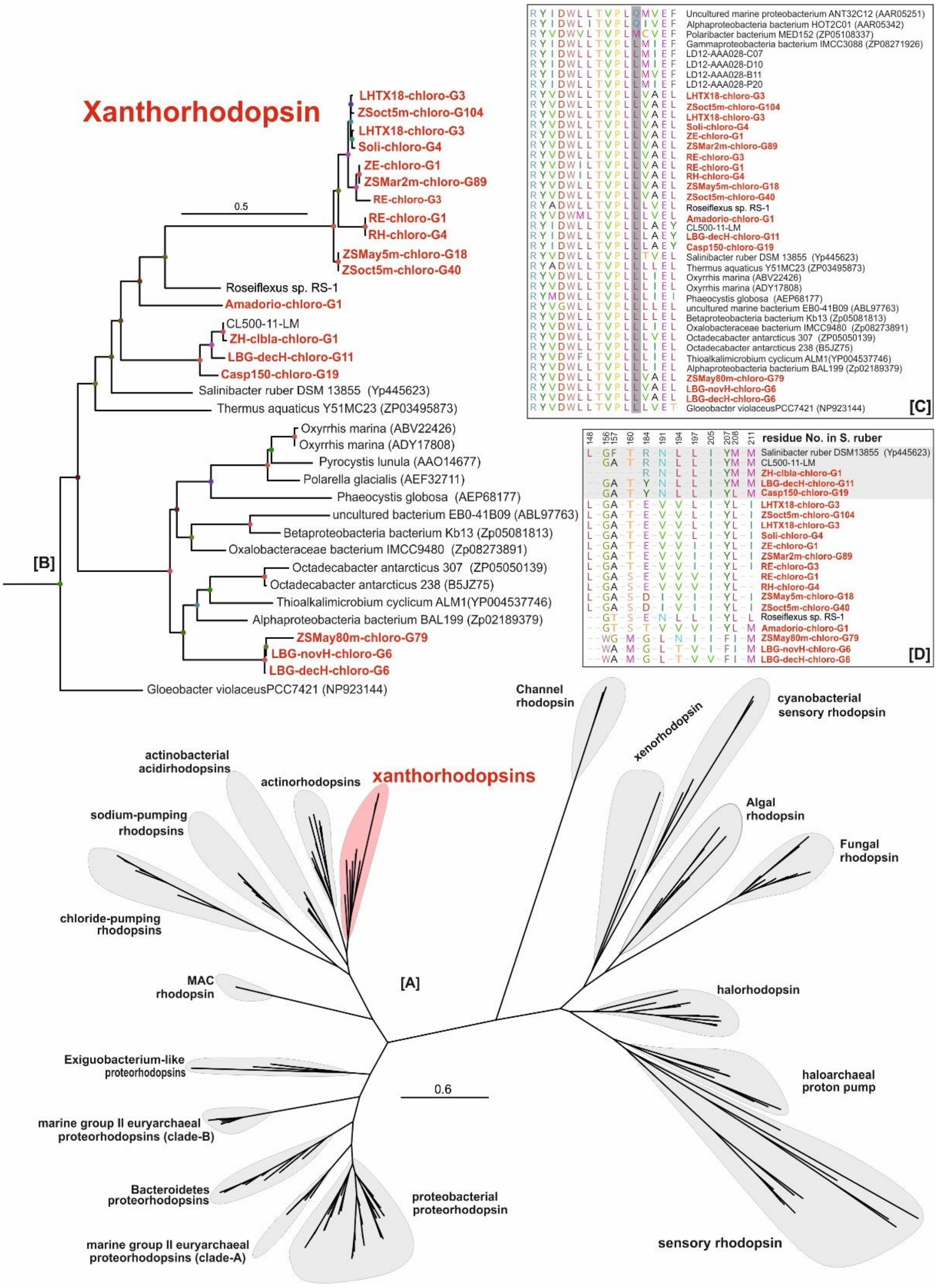

Supplementary Figure S8- Maximum likelihood tree of rhodopsin protein sequences from different bacterial and archaeal groups (212 protein sequences in total) [A]. Expanded Maximum likelihood tree of the rhodopsin protein sequences belonging to the phylum *Chloroflexi* [B]. The alignment of the rhodopsin protein sequences from the amino acid associated with light absorption preferences. The leucine (L) and methionine (M) variants absorb maximally in the green spectrum while the glutamine (Q) variant absorbs maximally in the blue spectrum [C]. The alignment of amino acid residues involved in carotenoid binding in Salinibacter ruber DSM13855 (Luecke *et al.*, 2008) and Xanthorhodopsin like sequences of the phylum *Chloroflexi*. The residue number is mentioned on top of the panel [D]. The rhodopsin genes present in the MAGs of this study are highlighted in re

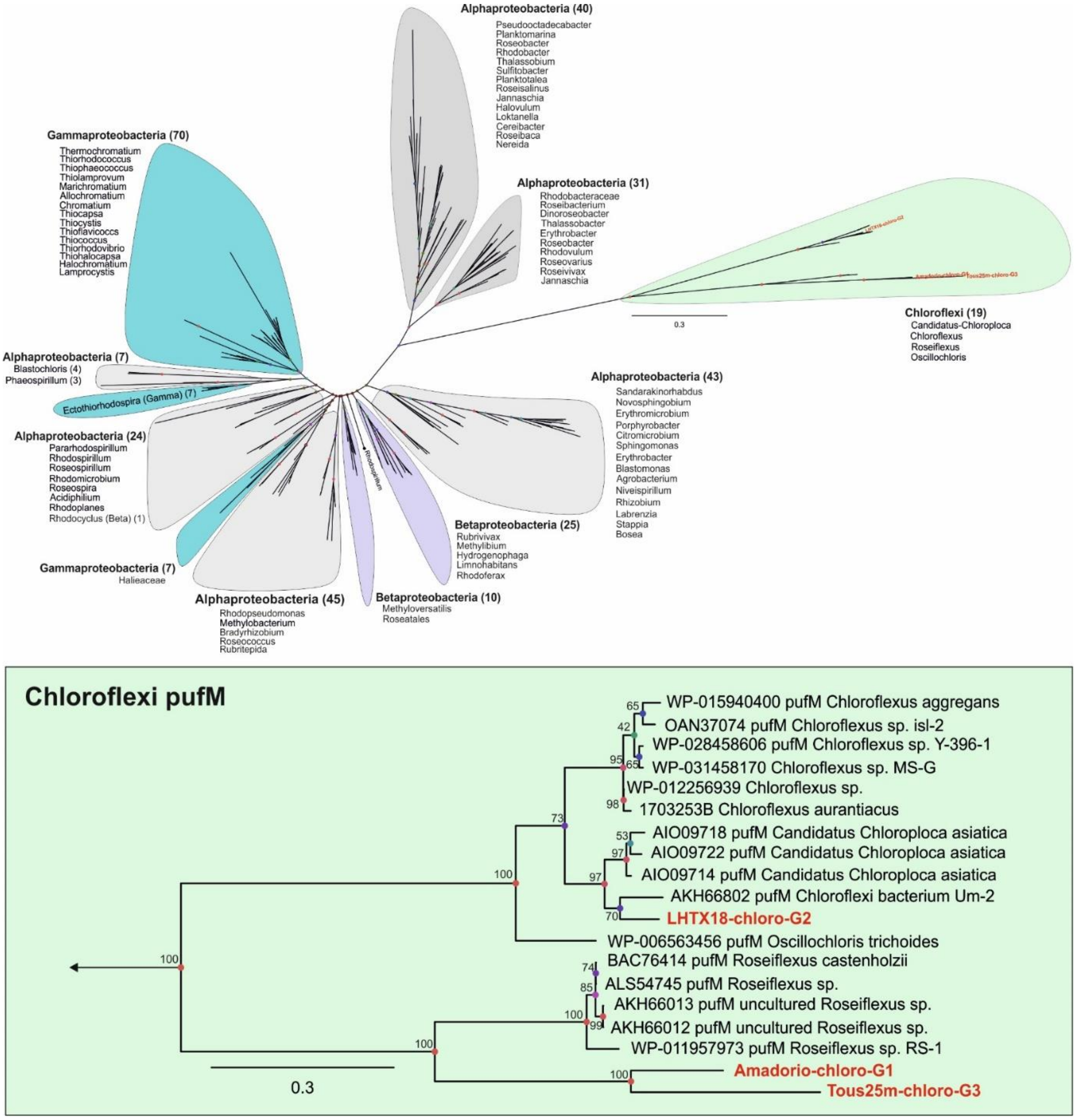

Supplementary Figure S9- Maximum likelihood tree of the *pufM* protein sequences from different bacterial groups (328 protein sequences in total) [A]. Expanded Maximum likelihood tree of the *pufM* protein sequences belonging to the phylum *Chloroflexi* [B]. The *pufM* genes present in the MAGs of this study are highlighted in red.
