# Supplementary material for: Hidden in plain sight—highly abundant and diverse planktonic freshwater Chloroflexi
Source: Microbiome. 2018 Oct 2;6:176. doi: 10.1186/s40168-018-0563-8 (PMC6169038; doi:10.1186/s40168-018-0563-8)
Supplement: Supplementary file 5 — Table S4. Details of CARD-FISH probes designed for specific Chloroflexi lineages. (PDF 107 kb) [file 40168_2018_563_MOESM5_ESM.pdf]

Table S4. Details of CARD-FISH probes designed for specific Chloroflexi lineages.

**probe name**	**target lineage**	**coverage**	**sequence (5'-3')**	**% FA**	**reference**
JG30-1027	JG30-KF-CM66 - freshwater branch	3/3	ATC TGT GAC ACA CGC CTT GCG G	40	this study
SL65-1025	SL56	45/45 (+168 reads)	GCA CGC GTC CCT TTC GGG A	60	this study
CL500-11	CL500-11 - freshwater branch	8/8 (+39 reads)	GCC GAC TTG CCC AAC CTC	40	Okazaki et al. 2013
TK10-345	TK10 - freshwater branch	28/28 (+14 reads)	CCT CCA TGC TAA ACC CCG	40	this study

For phylogeny of targeted lineages see Supplementary Figure S1. % FA, percentage of formamide in the hybridization buffer.
